# Do K-Pop Consumers’ Fandom Activities Affect Their Happiness, Listening Intention, and Loyalty?

**DOI:** 10.3390/bs14121136

**Published:** 2024-11-26

**Authors:** Hyun-ju Choi

**Affiliations:** Department of Cultural & Arts Management, Sangmyung University, Cheonan-si 31066, Chungcheongnam-do, Republic of Korea; hyunju_choi@naver.com

**Keywords:** online fandom activities, offline fandom activities, happiness, listening intention, loyalty, word of mouth/purchase, contemporary Christian music

## Abstract

This study examines the influence of K-pop consumers’ (online/offline) fandom activities on their happiness and their contemporary Christian music (CCM) listening intention and does so considering two base theories: activity theory and the content theory of motivation. In this context, we also examine the influence of happiness and CCM listening intention on CCM loyalty (word of mouth/purchase). We focus on global consumers of K-pop (people with experience in online/offline K-pop fandom activities) from two countries: the US and the UK. For our investigation, we surveyed these consumers between 1 April and 30 September 2022 through two global research agencies, namely Entrust Survey and META G DATA lnc. We received valid responses from 331 participants. We then used structural equation modeling to analyze the data and found the following: First, although K-pop consumers’ online fandom activities did not have a statistically significant effect on their happiness, their offline fandom activities did. Second, their fandom activities had a statistically significant positive effect on their CCM listening intention, although their offline fandom activities did not. Third, their happiness also had a statistically significant positive effect on their CCM listening intention. Ultimately, their happiness and CCM listening intention had a statistically significant positive effect on their loyalty (CCM word of mouth/purchase). We identified a new trend and applied it in the context of K-pop culture and CCM, thereby contributing to consumer psychology studies through creative/innovative empirical research.

## 1. Introduction

K-pop is a generic term for dance music, idol music, ballad music, soundtrack, etc. A more precise approach would be to analyze the lyrics’ characteristics, rather than the melody. Entertainment that caters to the needs of the global fandom is a crucial element. For example, the boy band BTS (Bangtansonyundan), has won over the world with their well-crafted message “Love Yourself”, which often comes across as a wholesome song [1,2,3,4,5].

K-pop and its stars are revered, particularly by teenagers and young adults, with their concerts likened to religious ceremonies and their concert venues to places of worship [6,7]. The epitome of this devotion is the “fandom” culture, a term derived from the word “fanatic”, denoting an individual with extreme enthusiasm for a specific entity or field, and the suffix “dom”, which signifies a territory or country. “Fandom” refers to people who exhibit excessive fervor for a particular person or field or for a cultural phenomenon of such nature [8,9]. K-pop idol fans actively engage in their favorite star’s life by posting about their daily activities on fan pages, sharing a variety of self-taken celebrity photos, known as *jikjjik* pictures, and closely monitoring their every move. They also read or write fan fiction, fictional works featuring their favorite star as the protagonist, and involve themselves in all aspects of their idol’s life. To these fans, their idols represent the “truth” and “light”, as well as the guiding “deity” of their lives [10,11].

The popularity of K-pop stems largely from the concept of a “united fandom culture [7,10]”. This refers to a sense of community among fans, or even between the fans and the star, fostered through close communication [7,10]. Owing to BTS, K-pop has expanded beyond Asia and into English-speaking countries (US/UK), and it is capturing people’s attention across the world. BTS has widened the K-pop market to another level, creating a significant milestone in K-pop history and fandom culture. A discussion about BTS (Bangtansonyundan), the world’s most renowned Korean boy band, cannot exclude the mention of ARMY, their official fandom. The fandom name comes from the English word “army”, signifying that BTS and its fans are always together, like an army and its body armor. ARMY shares a common fate with BTS, supporting them constantly and everywhere. Indeed, ARMY played a crucial role in drawing the attention of the media in the Anglosphere, previously perceived as an impenetrable fortress, to BTS. Anyone seeing this unique group, which spontaneously appears both online and offline chanting BTS’s name, would naturally be curious about who they are. [1,2,3,4,5]. The media in the Anglosphere were particularly intrigued by the question, “Where do these passionate and loyal individuals originate from?” K-pop fandoms, having evolved into their own culture through numerous transformations over approximately two decades, are unlike any other fan group worldwide. Their passion leans more towards self-sacrifice than self-destruction, and they value the strong connection they share with fellow fans of the same subject [12,13]. In particular, a major difference exists between the fandoms of K-pop and others. Specifically, K-pop fandoms seek meaning in the process of consuming K-pop music, exercising positive influence through fandom activities. K-pop fandoms have evolved into a highly active form of collective online behavior.

As previously discussed, fandoms have been central to this borderless globalization. K-pop fandoms, which initially drew global attention to K-pop and continue to expand, are projected to surpass 200 million participants worldwide by 2023 [14]. Therefore, fandoms are viewed as a key factor that distinguishes K-pop from other popular music genres. K-pop fans may initially be attracted to a group because of their music, but they often grow to appreciate the group’s character, or more specifically, that of a particular group member, more than the music itself. These fans then willingly exhibit an extraordinary level of dedication to both the member and the group [8,12].

K-pop fans, who come from various nationalities and races, establish strong friendships and form positive reciprocal relationships with their favorite stars through shared interests and affection. This is particularly evident during live concerts where fans show their support by waving light sticks and signs, creating a unity between the stars and their fans [1,2,3,4,5]. These global fans report experiencing a “sense of belonging”, “sense of closeness”, and “happiness” in this nurturing environment. They also demonstrate their love for K-pop by buying not only their favorite star’s albums, but also a variety of merchandise related to the star or brand [10,12]. Moreover, K-pop is not confined to a specific music genre.

Essentially, the structure and function of the K-pop fan community can be likened to a religious group. This comparison was proposed by Cavicchi [15,16], a cultural historian and a fan of the American rock singer Bruce Springsteen, during his study of the fan community of which he was a part. Cavicchi [15,16] suggested that being a fan of someone can often be a “religious experience”. Fan activities directed toward pop music stars provide fans with a collective identity and a sense of belonging that surpasses their personal identities. This allows them to experience a form of transcendence in their relationship with the stars they admire. Moreover, fans derive “joy” from their fan activities through their consistent efforts toward identity, sense of belonging, and transcendence [15,16].

For example, events such as concerts offer an experience akin to religious assemblies, immersing fans in a unique state. However, fan activities extend beyond just concerts. Similarly to how religious individuals pay close attention to religious music during moments of meditation or divine service, fans attentively listen to their favorite artist’s music when they can maintain high concentration [17,18,19,20]. They also express their identity by cutting out articles about the singers, collecting their photographs, creating and sharing their own mementos, and displaying various items (albums, posters, etc.) that represent the star in their private space. Fans discuss their favorite artist’s life and music in the same way believers debate theology and doctrines, and they incorporate aesthetic interpretations or link the artist’s personal life with their music, thereby enriching the interpretations [17,18,19,20].

Contemporary Christian music (CCM) is presently carving out a niche as a fresh genre among various popular music styles [21]. In Korea, it is often referred to as a “gospel song” [22]. CCM is a music field and is seen as a genre of modern popular music that either directly or indirectly communicates Christian beliefs through its lyrics. In the United States and Europe, CCM is already performing on par with mainstream pop music, even reaching the top of the pop charts. This indicates that songs expressing Christian sentiments, ethics, love, and social justice, which differ from mainstream pop, are gaining recognition in their own right [23,24].

Notably, the K-pop “idol” phenomenon is extending into the realm of CCM. K-pop idols engage with disadvantaged individuals in churches, prisons, and nursing homes to share the gospel. In essence, the heartfelt praise and donations from those expressing gratitude surpass the emotional impact of any renowned singer’s performance [25]. Above all, the most significant change is that anyone, whether religious or not, can reaffirm their reason for living, purpose, and sense of mission through CCM [6,12]. Furthermore, attempts are being made to incorporate CCM into all K-pop genres and broaden the ecosystem to promote K-CCM internationally [26]. These endeavors are anticipated to enhance the satisfaction of K-pop consumers and potentially increase their intention to listen to CCM [12,26].

Specifically, with the exception of a few K-pop fans, most embrace their preferred artists as a very normal part of their daily life, gaining psychological rewards, especially happiness, in the process. It is, therefore, necessary to first observe whether the fandom activities of K-pop consumers positively influence happiness in daily life. An empirical study is required to determine whether happiness, a parameter in this study, grows with increased participation in fandom activities. Furthermore, the independent variable of fandom activities should be examined on the basis of online and offline activities.

However, previous studies have not adequately explored empirical research related to the fandom activities, happiness, CCM listening intention, and CCM loyalty of K-pop consumers. In this study, CCM listening intention refers to listening to, listening to as a priority, often listening to, regularly listening to, and frequently listening to the CCMs of favorite K-pop singers. Additionally, there is a lack of research investigating the effect on CCM loyalty using the happiness and CCM listening intention of K-pop consumers as parameters. CCM loyalty in this study is defined as the passion/initiative to spread word-of-mouth about or purchase CCM products of the K-pop artists people like. As such, this study examines the effect of the K-pop idol syndrome and fandom activities, which have evolved to resemble a form of religion, on the happiness, CCM listening intention, and CCM loyalty of K-pop consumers. This study proposes the following research questions:

### Research Questions

RQ 1. How do the fandom activities of K-pop consumers affect their happiness and CCM listening intention?
RQ 2. How does the happiness of K-pop consumers affect their CCM listening intention and CCM loyalty?
RQ 3. How does the CCM listening intention of K-pop consumers affect CCM loyalty?

## 2. Literature Review

### 2.1. Base Theory

This study conducts in-depth empirical tests of the influence of K-pop fans’ activities on their happiness and their intention to listen to CCM. It uses two foundational theories, namely activity theory and the content theory of motivation, to investigate the effect of happiness and the intention to listen to CCM on CCM loyalty.

#### 2.1.1. Activity Theory

Activity theory examines the group characteristics of learning through a structured approach to human activity systems. Researchers such as Vygotsky initiated studies on this theory in the 1920s and 1930s, aiming to explain intricate human activities and social phenomena [27,28]. Activity theory posits that an individual’s engagement with the social environment, such as fandom activities, produces a mediating artifact, for instance, happiness. This artifact represents an externalized version of the individual that can be accessed and used by the individual or others, such as the intention to listen to CCM. This, in turn, can foster social exchange [29,30,31].

**Figure 1:**
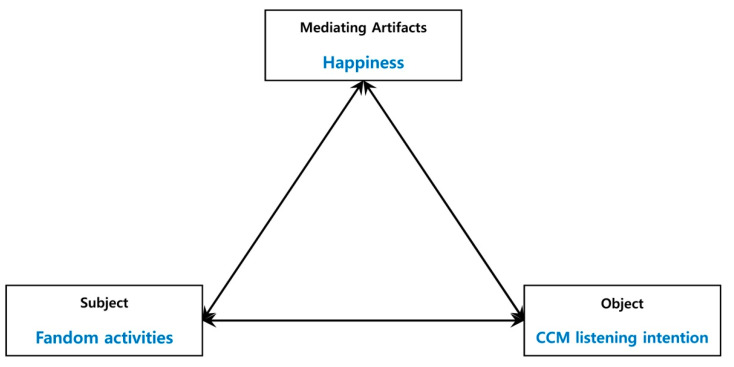
Activity system model.

Mediating artifacts are crucial in the context of activity theory [32]. For example, happiness, acting as a mediating artifact, might have a significant mediating role in the process where the subject, which in this case refers to the fandom activities of K-pop consumers, influences the object, which is the intention to listen to CCM [12,26,33,34]. What is important to note here is not just that mediators are capable of mediating but that cultural or social mediation creates the environment within which individuals live and operate and in which they can co-construct meaning with others about the actions of the mediator [32]. In other words, fandom activities (online/offline) of K-pop consumers may simply end up being just a hobby. However, these fans may seek happiness—a feeling of finding sufficient satisfaction and joy in daily life—through K-pop fandom activities. In short, such activities should be interpreted under a social context. Happiness from these activities acts as a mediating artifact in this study. In summary, individual behavior, such as fandom activities, can trigger happiness, which acts as a mediating artifact [10,35] (Figure 1).

Activity theory’s fundamental concept is the interaction between subject and object. This theory interprets activities within the subject–object relationship on three distinct levels: activity, action, and operation [27,36,37]. An action is a conscious process directed toward a goal that needs to be executed to fulfill a purpose. Various actions may be undertaken to reach this goal. On the other hand, an operation refers to a process carried out automatically, contingent on the action taken. In general, operations are immediate automated actions as well as habitual actions performed without conscious awareness [27,36,37]. For example, listening to a favorite K-pop artist’s CCM can be considered an operation. Importantly, the components of activity, as proposed by activity theory, are not static, but can change dynamically based on the situation [12,26].

Consumers of popular culture, when organized into fandoms, seek personal meaning through popular music. Fandoms act as a platform for projecting one’s identity and social imagination [9,38]. In this study, projection refers to the sharing and communication of an individual’s inclination to be part of a fandom, such as attitudes or traits. Consequently, fans use K-pop as a platform for group behavior, adapting it to their social characteristics, cultural landscape, and political issues [38]. This aspect is varied and bears similarities to a religious community or gathering, such as conflict or negotiation with cultural conservatism, resistance to political authoritarianism, or active endorsement of the country’s official nationalist agenda [39,40].

In summary, activity theory serves as a foundational theory for examining the causal relationship between the fandom activities of K-pop consumers (the independent variable) and happiness and CCM listening intention (the dependent variables). This establishes the progression from fandom activities to happiness and then to CCM listening intention.

#### 2.1.2. Content Theory of Motivation

Motivation suggests that a motive acts as a driving force behind human behavior and that there must be a motive to trigger such behavior, which stems from human autonomy. In essence, motivation involves exerting a significant amount of voluntary effort to reach objectives while gaining the capacity to fulfill personal needs [41]. The content theory of motivation investigates the inherent reasons for motivation in humans [42]. More precisely, this theory examines the primary elements of motivation and determines which of these factors affect individuals.

This study focuses primarily on the theory of achievement motivation, a part of the content theory of motivation. McClelland et al. [43] were the pioneers in introducing the concept of achievement motivation among many researchers studying motivation. For instance, when the theory of achievement motivation is applied to this research, several psychological factors are relevant when K-pop consumers participate in online or offline fandom activities with specific plans or goals [44]. Achievement motivation is not driven by external rewards, such as social honor and duty, but by a powerful motive, such as a passion or enthusiasm for K-pop, which provides joy and satisfaction in the activity itself, such as fandom activities.

The theory of achievement motivation suggests that once the needs for love and belonging are met, individuals seek to fulfill esteem needs, such as attaining social status, which, in turn, lead to the pursuit of self-actualization [45,46]. McClelland et al. [43] proposed that, similarly to needs, human motivation follows a sequential pattern. Specifically, the motivation for survival is associated with physiological and safety needs, while the motivation for belonging underlies the need for love and belonging. Furthermore, esteem needs are driven by a power motive, and self-actualization needs are linked with achievement motivation. Therefore, although achievement motivation is a distinct motive, it is considered a high-level motive [46,47].

Once K-pop fans have their love and belonging needs met by their preferred artists, they become more involved in fan activities, develop needs for social status, and ultimately experience joy from a sense of accomplishment. This progression towards happiness is in line with the need for achievement motivation [10,48,49,50]. Hence, K-pop fans’ love and belonging needs for their favorite artists stem from a desire to belong. This suggests that increased passion and enthusiasm through fan activities can lead to enhanced happiness [51]. Moreover, fans bonded by shared enthusiasm show high loyalty. In essence, they invest more time in consuming content such as records and merchandise, support their favorite K-pop artists financially, and promote them to others to increase their popularity [1,2,3]. In other words, fandom, displaying high loyalty, plays a significant role in business expansion. Take, for example, offline fandom activities—the ticket price of K-pop events increases due to the high production cost of concerts, including expenses for stage settings, videography, and lights. Nonetheless, because of the high loyalty of K-pop fandoms, fans will pay to buy any product if it is related to their preferred artists. This also extends to consuming content (e.g., albums and merchandise).

Fundamentally, the content theory of motivation serves as a foundational theory to examine the causality between the fandom activities of K-pop consumers (independent variable), happiness and CCM listening intention (parameters), and CCM loyalty (dependent variable), such as word of mouth and purchase. This establishes the path from fandom activities to happiness, to CCM listening intention, and finally to CCM loyalty.

### 2.2. Concepts of Variables and Previous Studies

#### 2.2.1. Fandom Activities and Happiness

The term “fandom” is a combination of the word “fanatic”, referring to an individual with extreme enthusiasm for something, and the suffix “-dom”, which signifies territory. Essentially, it denotes a group of fans who favor a specific star or genre. With the advent of television and the spread of popular culture, fandoms that gained social and cultural influence evolved into cultural leaders known as fandom culture [52]. As various K-pop idols continue to rise, there is a symbiotic relationship forming between fandom culture and these idol singers. Particularly since the 2000s, when fan club activities experienced a surge, the spread of the internet has led fan club members to appreciate the connection they have with each other and to maintain close relationships through both online and offline social activities [53,54].

Digital media serves as a bridge connecting various elements. For instance, amidst the COVID-19 pandemic, K-pop idols could not conduct live performances, yet their fan bases experienced substantial growth [55,56,57]. Despite the lack of offline performances, these idols maintained a frequent presence, not only in traditional media, but also on social media platforms. This visibility attracted new fans who had not previously witnessed their performances, leading to anticipation for their future shows. Consequently, the content generated by existing fans facilitated the expansion of these fandoms. In essence, current social media platforms have seamlessly integrated these idols into the global content distribution process [6,55].

With the lifting of COVID-19 social distancing measures, offline fan groups are exhibiting increased enthusiasm in their activities. Numerous fans are not only buying albums and streaming content continuously, but they’re also attending their favorite stars’ TV appearances or live radio broadcasts [9,11]. Fans also attend live concerts or music shows to support their idols, waving light sticks or balloons to demonstrate their fandom’s magnitude. The actions of these individual fans coalesce into a significant group activity, thereby exerting a substantial influence [10,48]. Furthermore, attending offline events often involves travel. During these journeys, fans, whether consciously or unconsciously, become acquainted with each other, providing opportunities for social interaction. This is particularly true in concert venues. Fans experience performances “together” with others who share the same interests. This sense of community persists even after the event. Consequently, fans often share their pre- and post-performance routines, even if these are not explicitly fan activities [11,49].

Prior research indicates that individuals derive a sense of joy from participating in fandom activities, which subsequently increases their interest in foreign languages or professions that provide opportunities to meet their preferred musicians. This interest also fuels their motivation to learn by examining their roles, and their happiness intensifies in interpersonal relationships through engaging in fandom activities with fellow fans [10,12,55].

This study identifies happiness as a positive sentiment characterized by hope, satisfaction, joy, and a sense of reward in daily life. It also denotes a psychological state where an individual feels satisfied, content, and relaxed because their needs and desires are fulfilled, and they experience minimal anxiety. This condition can be either subjective or objective [58,59].

As discussed previously, fandom culture offers a positive aspect by fostering a unique cultural community through active interaction and unity among fans [12,13,60]. Every fan involved in this cultural community is free and equal, experiencing the same sense of belonging and joy. In other words, all members of the fandom community share crucial information about their favorite stars, communicate on an equal basis, and derive pleasure [35,38]. Moreover, fandom activities enable fans to share their interests with others, and they feel joy when they are in social or personal spaces with others [58,59]. Therefore, based on the studies reviewed so far, this study establishes the following hypothesis:

**H1a.**  *K-pop consumers’ online fandom activities have a positive effect on their happiness.*

**H1b.**  *K-pop consumers’ offline fandom activities have a positive effect on their happiness.*

#### 2.2.2. Fandom Activities and CCM Listening Intention

Listening involves showing interest in what others are saying. It is not just about receiving a message through sight and hearing, but it also involves a specific response or attitude that interprets the message by discerning the thoughts or feelings behind it. Furthermore, listening encompasses a sequence of attitudes that begin with merely hearing the other person’s verbal expressions, and then responding to the message by empathizing with it [61,62].

CCM is adopting a contemporary music culture that resonates with individuals raised in a multicultural setting [63]. In particular, K-pop consumers are not hostile to the access to CCM. This demonstrates that CCM is now popular, and non-religious people can access it or listen to it without getting offended [25,64]. In this context, K-pop fans, who can now effortlessly enjoy K-pop music tracks or videos, are starting to feel the desire to participate actively [65,66]. They show enthusiastic support for their favorite groups, often referred to as “all-time favorites”, and buy special albums (such as CCM albums) not for listening, but for collecting or sponsoring [67]. They even partake in social activities or charity projects through the CCM of their favorite groups [68]. For example, a charity concert or bazaar to help the underprivileged through CCM performances with idol singers. This behavior is backed by the fact that CCM is driven by the Christian music industry and artists. Furthermore, K-pop fans recognize the value of CCM as a music product beyond its religious aspect, as evidenced by their purchasing or listening to CCM albums [69,70,71]. Therefore, based on the studies reviewed so far, this study proposes the following hypothesis:

**H2a.**  *K-pop consumers’ online fandom activities have a positive effect on their CCM listening intention.*

**H2b.**  *K-pop consumers’ offline fandom activities have a positive effect on their CCM listening intention.*

#### 2.2.3. Happiness and CCM Listening Intention, and Loyalty

Loyalty, for example, pertains to the frequency or extent of repurchasing a particular product. This type of loyalty manifests when an individual exhibits a high level of engagement with the product and makes relatively uncomplicated decisions [11,72,73]. Therefore, loyal consumers are essential in the K-pop industry because they generate profits for the company [11]. Concentrating on highly loyal consumers is a traditional marketing strategy. There are two types of loyalty: “behavioral loyalty”, which centers on consumer behavior patterns, and “emotional loyalty”, which emphasizes emotional connections with brands or services [11,74].

In simple terms, “behavioral loyalty” refers to the repetition of a specific goal, behavior, or conversion. This is an aspect of consumer behavior, such as the repeated purchase of a specific brand [75,76]. On the other hand, “emotional loyalty” is grounded in affection for and satisfaction with a brand, leading to a more enthusiastic and friendly approach to voluntary support activities and the intention to recommend to others as well as repeated conversion. Therefore, customers with emotional loyalty are often referred to as “hardcore fans”. It is crucial to enhance not just behavioral loyalty but also emotional loyalty for the growth of a service [77,78]. In this study, loyalty encompasses both behavioral and emotional loyalty.

Research indicates that human happiness is closely tied to interpersonal relationships [77,78]. Thus, when do people experience happiness? Experts suggest that empathy and active listening are crucial for emotional connection. Happiness can be found in daily life by simply being an attentive listener and empathizing with others rather than dominating the conversation [79,80,81]. Listening can vary in type and intensity, such as attentive listening in intimate relationships, casual listening with frequent acquaintances, or deferential listening as a follower [82,83]. Drawing on these studies, it can be inferred that the happiness of K-pop consumers may enhance their intention to engage in CCM listening (passion/enthusiasm).

In the realm of popular culture, fandoms represent a consumer base. The contemporary interpretation of an idol fandom is primarily shaped by passionate consumption [70,84]. This term denotes an active engagement in augmenting the value of digital products, extending beyond the mere acquisition of both tangible and intangible cultural products produced by idol groups. Thus, the most significant role and identity of fandoms lies in their function as consumers [85,86]. All activities of fandoms as consumers are carried out through individuals sharing similar emotions as well as knowledge and information about a specific subject. Furthermore, enhancing value within a fandom requires voluntary participation. Ultimately, fandom consumption activities not only boost the market value of a specific subject but also hold value as productive endeavors that broaden the reach of fandom culture [1,2,3].

In essence, the decision-makers within K-pop fandoms are consumers, and their empathy is influenced primarily by the community. This suggests that information about K-pop can be sought and obtained through various sources, such as friends, family, or media, which allows K-pop consumers to reaffirm their enjoyment and satisfaction [10,12]. Additionally, K-pop consumers who have reconfirmed their enjoyment and satisfaction make purchases and decide on a specific brand and engage in close interaction. Moreover, the loyalty associated with K-pop ultimately leads to the intention to support a specific brand, resulting in retention and repurchase with enhanced loyalty [1,2,3,5,87]. Therefore, based on the studies reviewed so far, this study proposes the following hypothesis:

**H3.**  *K-pop consumers’ happiness has a positive effect on their CCM listening intention.*

**H4.**  *K-pop consumers’ happiness has a positive effect on their CCM loyalty.*

**H5.**  *K-pop consumers’ CCM listening intention has a positive effect on their CCM loyalty.*

## 3. Methods

### 3.1. Research Model

**Figure 2:**
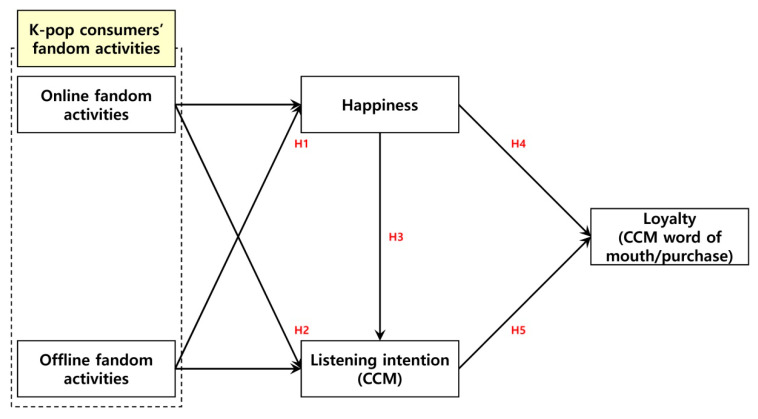
Research model.

Based on the theoretical concepts and extant research on the main variables examined earlier, we created a research model, as illustrated in Figure 2. Specifically, our model examines the influence of K-pop consumers’ (online/offline) fandom activities on their happiness and their contemporary Christian music (CCM) listening intention considering the two base theories: activity theory and the content theory of motivation. We also examined the influence of their happiness and their CCM listening intention on their CCM loyalty (word of mouth/purchase).

### 3.2. Variable Measurement

All of the variables assessed in our survey are based on extant literature and have been modified and supplemented according to our objective. We included 25 survey items for the variables, measured on a five-point Likert scale (where 1 = Not at all and 5 = Very much). Table 1 shows the specific survey measurements of our study.

### 3.3. Survey Respondents

We surveyed global K-pop consumers (people with experience in online/offline K-pop fandom activities) from two countries—the US and the UK—between 1 April and 30 September 2022 through the global research agencies Entrust Survey (http://entrustsurvey.com) and META G DATA lnc. (https://new.smartpanel.kr/main). We received valid responses from 331 consumers. Table 2 shows the demographics of these consumers.

### 3.4. Data Analysis

We used two statistical programs, SPSS (version 27, Chicago, IL, USA) and SmartPLS (version 3.3.9, Hamburg, Germany), to conduct our statistical analysis. First, we performed a frequency analysis to examine the consumers’ demographic characteristics. Next, we calculated the reliability using Cronbach’s alpha and performed factor analysis to test the validity of our metrics. Subsequently, we conducted correlation analysis to examine the closeness (i.e., correlation) of the variables. Finally, we used structural equation modeling (SEM) to analyze the causality between our key variables.

Thus, we tested our hypotheses using SEM analysis through the statistical program SmartPLS. To achieve this, we resampled 500 times using the bootstrapping technique. Bootstrapping is a non-parametric procedure that tests the statistical significance of various PLS-SEM model results, including path coefficients, Cronbach’s alpha, HTMT, and R^2^ values [1,2,3,4,5,72,92,93,94,95].

## 4. Results

### 4.1. Reliability and Validity

Table 3 shows the results of our reliability and validity tests for all our variables in relation to the survey items. We found that the Cronbach’s alpha for each variable was a minimum of 0.906, indicating high reliability. Additionally, the loading of each factor was a minimum of 0.778, indicating high validity, and the AVE was at least 0.727. Thus, we confirmed the strong reliability and validity of our study variables.

### 4.2. Correlation Analysis

Table 4 shows the results of our discriminant validity and correlation analyses. We examined whether the AVE square root exceeded the correlation coefficient for each variable. The results indicated that it did not exceed the correlation coefficient for any variable. Accordingly, we were able to confirm the discriminant validity of the variables in our study.

### 4.3. Hypothesis Tests

**Figure 3:**
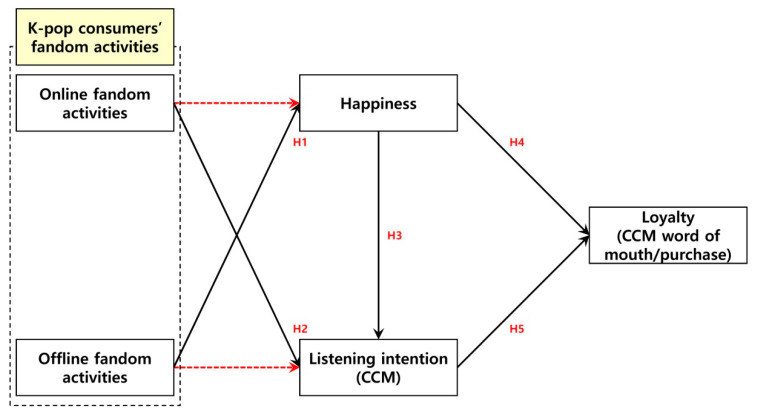
Hypothesis test results. Note: Solid black lines (statistically significant); Dotted red lines (not statistically significant).

Our study examined the influence of K-pop consumers’ (online/offline) fandom activities on their happiness and CCM listening intention. Additionally, we considered the influence of happiness and CCM listening intention on CCM loyalty (word of mouth/purchase). Our SEM analysis revealed the following:

First, K-pop consumers’ online fandom activities did not have a statistically significant effect on their happiness. However, their offline fandom activities did. Therefore, Hypothesis 1a was not supported, while Hypothesis 1b was. Second, K-pop consumers’ online fandom activities did have a statistically significant positive effect on their CCM listening intention. In contrast, their offline fandom activities did not. Therefore, Hypothesis 2a was supported, while Hypothesis 2b was not. Third, K-pop consumers’ happiness did have a statistically significant positive effect on their CCM listening intention. Therefore, Hypothesis 3 was supported. Finally, K-pop consumers’ happiness and their CCM listening intention both had a statistically significant positive effect on their loyalty (CCM word of mouth/purchase). Therefore, Hypotheses 4 and 5 were supported. Table 5 and Figure 3 present these results in detail.

### 4.4. Mediated Effect Test

We also analyzed whether the consumers’ happiness and CCM listening intention had a mediated effect on the relationship between their (online/offline) fandom activities and CCM loyalty (word of mouth/purchase). We found the following: First, consumer happiness and CCM listening intention did not show a mediated effect in the path of online fandom activities → happiness → CCM listening intention → CCM loyalty. Second, consumer happiness and CCM listening intention did show a mediated effect in the path of offline fandom activities → happiness → CCM listening intention → CCM loyalty. Table 6 presents these results in detail.

## 5. Discussion

### Summary of Results

Based on activity theory and content theory of motivation, our study empirically tested the effects of K-pop fans’ online/offline fandom activities on their happiness and CCM listening intention through structural equation model (SEM) analysis. We also tested the effect of happiness and CCM listening intention on CCM loyalty (word of mouth/purchase).

Our results showed that, first, the offline fandom activities of K-pop consumers had a statistically significant effect on happiness, whereas online fandom activities did not have such an effect. This result partially supports the results of previous studies [6,12,35,38,56,58,59]. Research has noted that the role of fandoms in everyday life has grown amid the massive culture shifts throughout the COVID-19 pandemic, political developments, and economic uncertainties. Fandoms provide people with a sense of security and confidence in their identities [6,48]. Six (64%) out of ten fans who participated in an Amazon Ads [96] custom research study (a survey of 12,000 respondents in Asia/Europe/America, etc.) responded that fandom is an element that determines their identity. Moreover, nearly half (48%) answered that fandoms help them understand the world. In the same survey, more than one-third (36%) of the fans responded that happiness is the biggest benefit they get as a fan. Therefore, K-pop consumers participating in offline fandoms can attempt a pleasant escape from everyday life and discover joy together, which may have significantly affected their happiness scores.

Second, the online fandom activities of K-pop consumers had a statistically significant positive effect on CCM listening intention, whereas offline fandom activities did not have such an effect. Namely this study finds the impact of offline fandom activities on CCM listening intention to be quite low. In other words, while CCM listening intention is strengthened through online fandom activities, impacting CCM loyalty, the influence of offline fandom activities is weak, and thus, does not affect CCM listening intention. This result partially supports the results of previous studies [69,70,71]. Consumers experience the positive effects of participating in fan culture and accept fandoms as a part of their lives [9,38]. This has helped fandoms become mainstream, providing new ways for fans to participate more often by finding like-minded fans on social media and building fan communities [6,10,12]. The composition of fandoms has also changed; virtually anyone can participate in fandoms regardless of age, gender identity, social/economic status, or any other characteristic [97]. Therefore, K-pop consumers participating in online fandoms are more likely to listen to content related to fandoms (e.g., CCM music) or consider using or purchasing relevant brands, which would explain the significant effects on CCM listening intention.

Third, the happiness of K-pop consumers had a statistically significant positive effect on CCM listening intention. This result supports the results of previous studies [98,99]. As fan culture has become more common and more ways to access fandoms have become available, a new kind of fan has emerged: fluid fans. Fluid fans consider themselves to be fans of any topic or brand they have passion for; they embrace a variety of fandoms [100,101]. Fluid fandoms are not necessarily superficial; they constantly explore the diversity existing in their fields of interest, and they consider fandoms as more than a hobby or a way to pass time. Fluid fans autonomously discover new content (e.g., CCM music) that brings them joy and feel happy about participating in it [9,10,12]. Therefore, K-pop fans strive to have deeper interactions and experiences with their favorite singers. In particular, since the music of their favorite K-pop singers helps convey the brand story to listeners throughout audio content overall, it may have significantly affected CCM listening intention.

Fourth, the happiness of K-pop consumers and CCM listening intention had a statistically significant positive effect on CCM loyalty (CCM word of mouth/purchase). As previously reported [1,2,3,5,87], fans and stars have a symbiotic relationship, which results in mutual happiness. Through stars, fans experience a kind of vicarious pleasure in which their dreams are fulfilled. Stars can also satisfy their needs for approval or achieve self-actualization through their fans. Thus, fans and stars become emotionally close and identify with each other [102,103]. Furthermore, fans imitate many aspects of their idols and are immersed in even the psychological aspect [10,104]. In sum, the happiness of fans is about fulfilling the nature of fandoms and satisfying a social need [105]. The happiness and belonging needs of fans, through fandoms, have evolved into a sense of accomplishment that makes fans feel they can create a specific culture [1,2,3,5,87]. This mechanism could explain the CCM listening intention we found. Therefore, fandoms represent power through fans. Happiness and CCM listening intention are important factors that can increase CCM loyalty.

## 6. Conclusions

### 6.1. Implications

#### 6.1.1. Theoretical Implications

First, our study is the empirical research to examine the effects of online/offline fandom activities on the happiness, CCM listening intention, and CCM loyalty (word of mouth/purchase) of K-pop consumers based on activity theory and the content theory of motivation.

Second, we identified a new trend and applied it in the context of K-pop culture and CCM, thereby contributing to consumer psychology studies through creative/innovative empirical research. Furthermore, this is the first study to apply the functions of a dialectical cycle between religion and popular culture as an organic reality through the convergence of humanities and business studies.

Third, this study conducted an empirical investigation employing the activity theory as the base theory. In other words, this paper empirically confirmed that the happiness of K-pop consumers enhanced with heightened passion/initiative towards their offline fandom activities. This supports the context of the activity theory. Therefore, offline fandom activities of K-pop consumers raise and help maintain happiness, i.e., a feeling of possessing enough satisfaction and joy in daily life. Furthermore, since it also raises the passion/initiative for the CCM listening intention of one’s preferred K-pop artists, the findings of this study make theoretical contributions to behavioral science and cultural sociology.

Fifth, the success stories of K-pop idols provide useful guidelines in terms of global missionary work and communication with Generations Y and Z, who are familiar with new digital technologies. Moreover, the perceived value of fans could be indirectly identified from the artistic aspect of religion, based on the use of idolization of stars, lyrics, melody, rhythm, and chords in Christian merchandise (e.g., CCM albums). Therefore, CCM can form social connections among K-pop consumers and function as a missionary tool.

#### 6.1.2. Practical Implications

First, this study confirms that fandom activities have expanded beyond the practice of personal taste to functioning as social leisure. Until now, fandom has been considered a highly personal hobby of enthusiasts, rather than a means to foster social connections. This study, however, found that the fandom activities of K-pop consumers provide happiness as social leisure, raise the CCM listening intention of their preferred K-pop artists, and even strengthen loyalty towards CCM. Based on this, the industry and the public need to recognize that fandom activities go beyond making K-pop artists successful and are a means to increase the value of fans’ lives.

Second, it is time for quality Christian pop culture content to be created by tapping into the specialized resources of Gen MZ and the next generation of singers, songwriters, composers, content planners, and developers. Moreover, Christian groups can develop a route where missionaries actively utilize K-pop popular culture content created through professional resources and provide a venue where such content can have a far-reaching impact. The K-pop craze, which is attracting global attention, can be a channel for missionary work. Also, it is necessary to make efforts so that CCM does not become one-off cultural content.

Third, missionary work can learn from the fandom activities of K-pop consumers while also seeking to overcome the following shortcomings: (1) Currently, K-pop idol singers communicate via two-way communication, namely entertainment company (record label) ↔ idols ↔ fans. Owing to technological advances in the digital age, K-pop idol singers typically use live-streaming platforms including YouTube. Furthermore, various social-media platforms, such as Instagram and Facebook, have emerged and strengthened direct communication between K-pop idol singers and their fans. These platforms have become an important medium for K-pop idol singers to share their personal moments with their fans, which can be viewed as a result of advances in digital technology and the evolving demands of fans. Therefore, similar to the case of K-pop idol singers, missionary activities based on contemporary Christian music should be actively broadcast using live-streaming platforms such as YouTube. (2) The Korean wave and the increasing popularity of K-pop necessitates the active framing of K-CCM as constituting the Korean wave. For instance, more cultural spaces based on the concept of Christian multicafe should be created, which allows people to worship as well as drink coffee and listen to CCM. Therefore, the globalization vision of K-CCM must be realized by identifying talented musicians and artists with passion and vision for Christian music.

Fourth, contemporary Christian music (CCM) performed by K-pop idol singers offers various advantages beyond simply increasing the appeal of popular music and religion. Idol singers, with their mass appeal and attractive public image, actively participate in diverse genres such as musicals, plays, and films in addition to their performances as singers. This versatility allows CCM, through idol singers, to achieve broader dissemination via mass media. Also, the empirical findings of this study suggest that religious music performed by K-pop idol singers can facilitate the creation of religious and cultural content in other faiths, such as Catholicism and Buddhism. In other words, the study underscores that the most critical factor in creating religious and cultural content is its popularization. This indicates that religious and cultural content should cater to both people of faith and the general public. Therefore, cultivating and expanding such content to broaden and popularize religious culture is crucial and demands continued attention moving forward.

### 6.2. Limitations and Future Research Direction

Currently, BTS has many fans across the globe. Fans use convenient streaming services to listen to music while also purchasing physical albums to collect artists’ photographs and merchandise. Moreover, fans tend to continue consuming albums based on trust in the companies that they have experience consuming before or that represent the artists whose value they appreciate [1,2,3,4,5]. The tastes of fans consuming music content sensitively respond to fads or trends of the generation. Accordingly, future work should more specifically examine content that combines K-pop and CCM. Furthermore, there is a need to explore various antecedent factors that can help CCM increase its fandom activities and loyalty among K-pop consumers through continuous transformations from a Christian perspective.

Consumers can gain different benefits from participating in fan culture and fan communities. In other words, fandoms of various categories provide different emotional and social benefits depending on the type of content or product characteristics on which each fandom focuses [9,38,54]. For instance, music increases self-esteem and emotional bond and provides the joy of content creation/discovery. Moreover, music fans have the rewarding experience of having fun, gaining confidence, and finding new content they can enjoy [9,10,12,50,52]. Therefore, future research should comparatively analyze the characteristics of K-CCM and K-pop as well as examine what aspects the fans of each fandom are most excited about, how casual fans participate in fandoms, what the latest trends in fandoms are, and how much money fans spend on their fandom.

## Figures and Tables

**Table 1:** Survey items for the variables.

Variable	Operational Definition	Measurement Item	Researcher
Online fandom activities	Favorite K-pop singer—Degree of online fandom activities (passion/initiative)	1. Favorite K-pop singer—I visit their homepage (including SNS).	An et al. [88]
		2. Favorite K-pop singer—I join fan club(s) or fan café(s).	
		3. Favorite K-pop singer—I write email(s) or fan letter(s).	
		4. Favorite K-pop singer—I comment on portals or articles.	
		5. Favorite K-pop singer—I upload photos/videos online.	
Offline fandom activities	Favorite K-pop singer—Degree of offline fandom activities (passion/initiative)	1. Favorite K-pop singer—I attend fan meeting(s).	An et al. [88]
		2. Favorite K-pop singer—I attend fan sign event(s).	
		3. Favorite K-pop singer—I go to a broadcasting station (to watch them).	
		4. Favorite K-pop singer—I go to concert(s).	
		5. Favorite K-pop singer—I go to music festival(s).	
Happiness	Degree of satisfaction, enjoyment, and sense of reward in daily life	1. Last 6 months—I am happy with my family life in general.	Shin [89]
		2. Last 6 months—I am happy with the household income in general.	
		3. Last 6 months—I am happy with my relationships in general.	
		4. Last 6 months—I am happy with my health in general.	
		5. Last 6 months—I am happy with my work/studies, etc. in general.	
Listening intention (CCM)	Favorite K-pop singer—Degree of CCM listening intention (passion/initiative)	1. Favorite K-pop singer CCM—I will definitely listen to it.	Yu [90]
		2. Favorite K-pop singer CCM—I will listen to it before listening to anything else.	
		3. Favorite K-pop singer CCM—I will listen to it often.	
		4. Favorite K-pop singer CCM—I will listen to it regularly.	
		5. Favorite K-pop singer CCM—I will listen to it occasionally.	
Loyalty (CCM word of mouth/purchase)	Favorite K-pop singer CCM goods (products)—Degree of word of mouth/purchase (passion/initiative)	1. Favorite K-pop singer CCM products (merchandise)—I positively consider buying them.	Kwon et al. [73] Lee & Kim [5] Lee & Kim [91]
		2. Favorite K-pop singer CCM products (merchandise)—I buy them even if the price is high.	
		3. Favorite K-pop singer CCM products (merchandise)—I buy them whether they are sold online or offline.	
		4. Favorite K-pop singer CCM products (merchandise)—I strongly recommend them.	
		5. Favorite K-pop singer CCM products (merchandise)—I talk about the purchase favorably.	

**Table 2:** Demographic characteristics (N = 331).

Item		Frequency	%
Gender	Male	165	49.8
	Female	166	50.2
Age	20 s	86	26.0
	30 s	96	29.0
	40 s	75	22.7
	50 s	74	22.4
Education	High school graduate	163	49.2
	Junior college graduate	34	10.3
	College graduate	107	32.3
	Graduate school graduate	27	8.2
Monthly income (Mean)	Under $1000	60	18.1
	$1000–$2000	95	28.7
	$2001–$3000	46	13.9
	$3001–$4000	44	13.3
	$4001–$5000	27	8.2
	$5000 over	59	17.8
Nationality	The U.S.	165	49.8
	The UK	166	50.2

**Table 3:** Reliability and validity.

Variable	Item	Convergent Validity			Cronbach’s Alpha
		Outer Loadings	Composite Reliability	AVE	
Online fandom activities	Online1	0.902	0.950	0.791	0.934
	Online2	0.922			
	Online3	0.869			
	Online4	0.890			
	Online5	0.862			
Offline fandom activities	Offline1	0.890	0.956	0.811	0.942
	Offline2	0.943			
	Offline3	0.896			
	Offline4	0.881			
	Offline5	0.893			
Happiness	Happiness1	0.818	0.930	0.727	0.906
	Happiness2	0.848			
	Happiness3	0.866			
	Happiness4	0.852			
	Happiness5	0.879			
Listening intention (CCM)	Listening intention1	0.900	0.946	0.779	0.928
	Listening intention2	0.917			
	Listening intention3	0.935			
	Listening intention4	0.873			
	Listening intention5	0.778			
Loyalty (CCM word of mouth/purchase)	Loyalty1	0.911	0.964	0.841	0.953
	Loyalty2	0.901			
	Loyalty3	0.926			
	Loyalty4	0.916			
	Loyalty5	0.930			

Note: Outer loadings > 0.70; composite reliability > 0.70; average variance extracted (AVE) > 0.5; Cronbach’s alpha > 0.70.

**Table 4:** Correlation analysis.

Variable		1	2	3	4	5
1	Online fandom activities	**0.889**	-	-	-	-
2	Offline fandom activities	0.840	**0.901**	-	-	-
3	Happiness	0.387	0.465	**0.853**	-	-
4	Listening intention (CCM)	0.805	0.724	0.496	**0.882**	-
5	Loyalty (CCM word of mouth/purchase)	0.765	0.746	0.507	0.848	**0.917**

Note: Boldfaced diagonal values indicate the square root of the AVE.

**Table 5:** Hypothesis test results.

Hypothesis Path				β	Sample Mean	Standard Deviation	t	*p*	Result
H1a	Online fandom activities	→	Happiness	−0.011	0.002	0.129	0.083	0.934	Not supported
H1b	Offline fandom activities	→	Happiness	0.474	0.460	0.121	3.929	0.000	Supported
H2a	Online fandom activities	→	Listening intention	0.672	0.678	0.082	8.152	0.000	Supported
H2b	Offline fandom activities	→	Listening intention	0.063	0.055	0.089	0.704	0.482	Not supported
H3	Happiness	→	Listening intention	0.207	0.209	0.052	4.013	0.000	Supported
H4	Happiness	→	Loyalty	0.094	0.093	0.030	3.148	0.002	Supported
H5	Listening intention	→	Loyalty	0.832	0.831	0.033	25.480	0.000	Supported

Note: R^2^ values: happiness = 0.216, listening intention = 0.690, loyalty = 0.778; model fit: SRMR = 0.049, NFI = 0.827.

**Table 6:** Mediated effect test results.

Path		β	Sample Mean	Standard Deviation	t	*p*	Mediated Effect
1	Online fandom activities → happiness → CCM listening intention → CCM loyalty	−0.002	0.000	0.022	0.082	0.934	No
2	Offline fandom activities → happiness → CCM listening intention → CCM loyalty	0.082	0.080	0.030	2.682	0.008	Yes

## Data Availability

Data sharing is not applicable. The data are not publicly available due to participant privacy.
